# Hyperglycemia in pregnancy did not worsen the short-term outcomes of very preterm infants: a propensity score matching study

**DOI:** 10.3389/fped.2024.1341221

**Published:** 2024-03-06

**Authors:** Ying Li, Wei Shen, Rong Zhang, Jian Mao, Ling Liu, Yan-Mei Chang, Xiu-Zhen Ye, Yin-Ping Qiu, Li Ma, Rui Cheng, Hui Wu, Dong-Mei Chen, Ling Chen, Ping Xu, Hua Mei, San-Nan Wang, Fa-Lin Xu, Rong Ju, Xiao-Mei Tong, Xin-Zhu Lin, Fan Wu

**Affiliations:** ^1^ Department of Neonatology, Guangzhou Key Laboratory of Neonatal Intestinal Diseases, The Third Affiliated Hospital of Guangzhou Medical University, Guangzhou, Guangdong, China; ^2^ Department of Neonatology, Women and Children’s Hospital, School of Medicine, Xiamen University, Xiamen, Fujian, China; ^3^ Xiamen Key Laboratory of Perinatal-Neonatal Infection, Xiamen, Fujian, China; ^4^ Department of Neonatology, Children’s Hospital of Fudan University, Shanghai, China; ^5^ Department of Pediatrics, Shengjing Hospital of China Medical University, Shenyang, Liaoning, China; ^6^ Department of Neonatology, Guiyang Maternal and Child Health Hospital·Guiyang Children’s Hospital, Guiyang, Guizhou, China; ^7^ Department of Pediatrics, Peking University Third Hospital, Beijing, China; ^8^ Department of Neonatology, Guangdong Province Maternal and Children’s Hospital, Guangzhou, Guangdong, China; ^9^ Department of Neonatology, General Hospital of Ningxia Medical University, Yinchuan, Ningxia, China; ^10^ Department of Neonatology, Children’s Hospital of Hebei Province, Shijiazhuang, Hebei, China; ^11^ Department of Neonatology, Children’ Hospital of Nanjing Medical University, Nanjing, Jiangsu, China; ^12^ Department of Neonatology, The First Hospital of Jilin University, Changchun, Jilin, China; ^13^ Department of Neonatology, Quanzhou Maternity and Children’s Hospital, Quanzhou, Fujian, China; ^14^ Department of Pediatrics, Tongji Hospital, Tongji Medical College, Huazhong University of Science and Technology, Wuhan, Hubei, China; ^15^ Department of Neonatology, Liaocheng People’s Hospital, Liaocheng, Shandong, China; ^16^ Department of Neonatology, The Affiliate Hospital of Inner Mongolia Medical University, Hohhot, Inner Mongolia, China; ^17^ Department of Neonatology, Suzhou Municipal Hospital, Suzhou, Jiangsu, China; ^18^ Department of Neonatology, The Third Affiliated Hospital of Zhengzhou University, Zhengzhou, Henan, China; ^19^ Department of Neonatology, Chengdu Women’ and Children’s Central Hospital, School of Medicine, University of Electronic Science and Technology of China, Chengdu, Sichuan, China; ^20^ Guangdong Provincial Key Laboratory of Major Obstetric Diseases, Guangdong Provincial Clinical Research Center for Obstetrics and Gynecology, The Third Affiliated Hospital of Guangzhou Medical University, Guangzhou, Guangdong, China

**Keywords:** hyperglycemia in pregnancy, preterm infant, outcomes, complication, growth retardation

## Abstract

**Background:**

Hyperglycemia in pregnancy (HGP) has generally been considered a risk factor associated with adverse outcomes in offspring, but its impact on the short-term outcomes of very preterm infants remains unclear.

**Methods:**

A secondary analysis was performed based on clinical data collected prospectively from 28 hospitals in seven regions of China from September 2019 to December 2020. According to maternal HGP, all infants were divided into the HGP group or the non-HGP group. A propensity score matching analysis was used to adjust for confounding factors, including gestational age, twin or multiple births, sex, antenatal steroid administration, delivery mode and hypertensive disorders of pregnancy. The main complications and the short-term growth status during hospitalization were evaluated in the HGP and non-HGP groups.

**Results:**

A total of 2,514 infants were eligible for analysis. After matching, there were 437 infants in the HGP group and 874 infants in the non-HGP group. There was no significant difference between the two groups in main complications including respiratory distress syndrome, bronchopulmonary dysplasia, necrotizing enterocolitis, retinopathy of prematurity, patent ductus arteriosus, culture positive sepsis, intraventricular hemorrhage, periventricular leukomalacia, anemia, feeding intolerance, metabolic bone disease of prematurity, or parenteral nutrition-associated cholestasis. The incidences of extrauterine growth retardation and increased growth retardation for weight and head circumference in the non-HGP group were all higher than those in the HGP group after matching (*P* < 0.05).

**Conclusions:**

HGP did not worsen the short-term outcomes of the surviving very preterm infants, as it did not lead to a higher risk of the main neonatal complications, and the infants’ growth improved during hospitalization.

## 1 Introduction

Hyperglycemia in pregnancy (HGP) is a maternal metabolic disorder, and its incidence is increasing worldwide. It includes two conditions known as gestational diabetes mellitus (GDM) and pregestational diabetes mellitus (PGDM), which are defined as abnormal glucose tolerance first found during pregnancy or diabetes before pregnancy, respectively. Some studies noted that HGP was associated with increased adverse effects on fetuses and infants, such as intrauterine hypoxia, preterm birth, birth injury, neonatal hypoglycemia, respiratory distress, and hyperbilirubinemia (1, 2). In addition, HGP can cause fetal overgrowth or backwardness *in utero* and increase the occurrence of macrosomia or small for gestational age (SGA) (3). These changes in the growth trajectory and metabolic level in infancy can even increase the risk of obesity and neurological damage in childhood or adulthood (4–7). Therefore, HGP has been regarded as a high-risk factor for short- and long-term adverse outcomes in offspring.

However, the impact of HGP on neonatal complications of very preterm infants (VPIs, gestational age <32 weeks) remains uncertain. There are some conflicting results in previous studies. Boghossian NS et al. (8) found that extremely preterm infants born to insulin-dependent diabetic mothers had higher risks of necrotizing enterocolitis (NEC), sepsis, and small head circumference but did not have an increased risk of patent ductus arteriosus (PDA), intraventricular hemorrhage (IVH), periventricular leukomalacia (PVL), or bronchopulmonary dysplasia (BPD). Grandi C et al. (9) found that only NEC was significantly higher in very low birth weight infants born to mothers with HGP. Opara CN et al. (10) noted that HGP was associated with an increased incidence and severity of retinopathy of prematurity (ROP). In contrast, other reports indicated that HGP did not lead to an elevated risk of in-hospital mortality or severe morbidity in preterm infants, including respiratory distress syndrome (RDS), severe IVH (grade 3–4), PDA treatment, ROP treatment, BPD or NEC (11–13). These controversial results may be partly attributed to the differences in the characteristics of the study populations and diagnostic criteria for HGP.

Additionally, the influence of HGP on the growth of VPIs should be assessed in more detail. Previous studies have found maternal early-pregnancy blood glucose levels are associated with altered fetal growth patterns, characterized by decreased fetal growth rates in mid-pregnancy and increased fetal growth rates from late pregnancy onward, which indicate a limited effect of increased fetal growth rates in fetuses <32 weeks (14). In contrast, they are more prone to extrauterine growth retardation (EUGR) due to immaturity and various complications after birth (15). Severe EUGR will negatively impact the growth potential of infants (16).

Therefore, it is necessary to further identify the impact of HGP on neonatal complications and growth in VPIs. In this study, to minimize the differences in the study population and confounding factors, we analyzed the clinical data of VPIs from 28 hospitals in seven regions of China using propensity score matching (PSM) analysis.

## 2 Materials and methods

### 2.1 Study population

This was a secondary analysis of clinical data collected by the Nutritional Committee of Neonatology Branch of Chinese Medical Doctor Association, National Multicenter EUGR Collaborative Group. The collaborative group was founded in 2019 to investigate the incidence and risk factors for EUGR in VPIs during hospitalization in different regions of mainland China (17). The study protocol was approved by the Ethics Committee of the Women and Children's Hospital Affiliated to Xiamen University/Xiamen Maternity and Child Health Care Hospital (KY-2019-016). It was registered at http://www.chictr.org.cn, and the registration number was ChiCTR1900023418.

The inclusion criteria were as follows: (1) gestational age <32 weeks; (2) admission within 24 h after birth; and (3) a hospitalization stay ≥2 weeks. The exclusion criteria were as follows: (1) severe congenital malformations or inherited metabolic diseases; (2) in-hospital death, treatment interruption or automatic discharge; and (3) incomplete or missing data. The criteria for discharge were as follows: (1) a weight of 1 800–2,000 g or more; (2) a corrected age ≥36 weeks; (3) cured primary disease and stable vital signs (infants with BPD were allowed to be discharged with oxygen); and (4) oral feeding, with a milk volume reaching full enteral feeding. Finally, 2,514 infants were included in the analysis (17). In this study, they were divided into the HGP or non-HGP groups according to whether the mother had HGP or not.

### 2.2 Data collection

From September 2019 to December 2020, the clinical data of VPIs admitted to the neonatal intensive care unit (NICU) of the 28 included hospitals located in seven regions of mainland China were collected prospectively, including four maternal/child health centers, eleven children's hospitals and thirteen tertiary general hospitals. According to a unified questionnaire, the general clinical data of maternal and pregnancy disorders, perinatal conditions, neonatal growth, nutritional support during hospitalization, neonatal complications and main treatments were collected. To minimize bias among hospitals and investigators, comprehensive and systematic training was provided to all the staff involved in the survey. Data collected by the researcher at each collaborative NICU were supervised and checked by the NICU director, who was responsible for quality assurance.

### 2.3 Definitions and classifications

The diagnostic criteria of HGP referred to the guidelines issued by the WHO in 2013 (18). Intrauterine growth retardation (IUGR) referred to a birth weight, length or head circumference below the 10th percentile of the growth curve of infants with the same sex and gestational age (19). The diagnostic criteria of the main complications were the same as those previously described (17). Briefly, neonatal RDS was diagnosed in preterm infants with respiratory distress shortly after birth. The criterion for grading RDS was based on the chest x-ray, and grade 3–4 indicating severe RDS. BPD was defined as oxygen dependence for at least 28 days, and moderate to severe BPD was defined as the need for oxygen therapy, positive pressure ventilation or mechanical ventilation at the corrected age of 36 weeks or at discharge. The diagnosis and grading of NEC were defined according to the modified Bell criteria. The diagnosis of early-onset sepsis (EOS) or late-onset sepsis (LOS) was defined as clinical symptoms before or after 72 h of admission, with or without positive cultures from blood or cerebrospinal fluid samples. PDA was diagnosed by echocardiography after 72 h of admission, and hemodynamically significant PDA (hsPDA) was defined as an arterial duct diameter >1.5 mm, a left atrial diameter/aortic diameter ≥1.4 or a left ventricular end-diastolic diameter/aortic diameter ≥2.1 accompanied by one of the following clinical manifestations: heart murmur, tachycardia (sustained ≥160 beats/min), increased breathing, increased pulse pressure (>25 mm Hg), hypotension, water pulse, or cardiac enlargement. Treated PDA referred to hsPDA requiring fluid intake limitation, the administration of diuretics, ibuprofen, or acetaminophen, or surgical ligation. ROP and its grades were defined by the international classification of ROP. ROP treatment referred to intravitreal drug injection, laser therapy or surgery intervention. Both IVH and PVL were diagnosed by cranial ultrasonography or magnetic resonance imaging (MRI). Papile's criterion was used to grade IVH, and grades 3–4 was regarded as severe IVH. PVL was defined as the degeneration of white matter adjacent to the cerebral ventricles following cerebral hypoxia or brain ischemia. Anemia was defined as a hemoglobin (Hb) level ≤130 g/L in venous blood or ≤145 g/L in capillary blood within 2 weeks after birth and an Hb level ≤100 g/L in venous blood or ≤110 g/L in capillary blood two weeks later. EUGR referred to a weight, length or head circumference at 36 weeks of corrected age or at discharge that was below the 10th percentile according to the Fenton growth chart for preterm infants. Feeding intolerance (FI) was defined as gastric retention up to 25%−50% of the previous feeding amount, abdominal distension or bloody stool, vomiting or bile reflux after repeated feeding, or coffee-like substances in the stomach. Metabolic bone disease of prematurity (MBDP) was defined as a serum alkaline phosphatase level >900 IU/L, accompanied by a serum phosphorus level <1.8 mmol/L. Parenteral nutrition-associated cholestasis (PNAC) was defined as conjugated bilirubin levels >1.5 mg/dl (25 μmol/L) at 2 consecutive measurements by spectrophotometric quantitation, followed by parenteral nutrition for more than 14 days and the exclusion of other diseases. Total enteral feeding was defined as an enteral feeding amount reaching 150 ml/(kg·d) or a total calorie intake reaching 110 kcal/(kg·d). Growth velocity (GV) was calculated as follows: GV[g/(kg·d)]^ ^= [1,000×ln(Wn/W1)]/(Dn-D1), where Wn is the discharge weight (g), W1 is the birth weight (g), Dn is the length of hospital stay (d), and D1 is the time to return to birth weight (d).

### 2.4 Statistical analysis

All data were analyzed using SPSS 26.0 software (IBM, Armonk, NY, USA). We conducted a 1:2 matched analysis by PSM with a nearest-neighbor matching algorithm to adjust for differences in baseline characteristics between the two groups, including gestational age, twin or multiple births, sex, antenatal steroid administration, delivery mode and hypertensive disorders in pregnancy. These covariates were selected based on reported studies (15, 20). Continuous variables are expressed as the mean ± standard deviation (*SD*) or median (*IQR*) according to whether the distribution was skewed and were analyzed using a *t test* or *Mann–Whitney test*. Categorical variables are expressed as frequencies (%) and were analyzed using *Pearson's chi-square test*. *P* < 0.05 was considered statistically significant.

## 3 Results

### 3.1 General characteristics

In total, 2,514 infants were eligible for statistical analysis (17). Before matching, there were 437 infants in the HGP group and 2,077 infants in the non-HGP group; after matching, the numbers of infants in the two groups were 437 and 874, respectively. The population characteristics of the two groups after matching are shown in Table 1. After matching, all six covariates were well balanced, with no significant differences between the two groups.

**Table 1:** The population characteristics between the HGP group and the non-HGP group.

Characteristics	HGP (No. = 437)	Non-HGP (No. = 874)	*P* value
Matching factors			
Gestational age, weeks	29.71 ± 1.49	29.71 ± 1.57	0.970
<28 weeks	61 (14.0)	120 (13.7)	0.910
28–31 weeks	376 (86.0)	754 (86.3)	
Sex			
Male	243 (55.6)	473 (54.1)	0.610
Female	194 (44.4)	401 (45.9)	
Twin or multiple birth	177 (40.5)	348 (39.8)	0.811
Antenatal steroids administration			
Total	338 (77.3)	676 (77.3)	>0.999
Partial course	121 (27.7)	232 (26.5)	0.660
Complete course	217 (49.7)	444 (50.8)	0.696
Mothers with HDP	60 (13.7)	118 (13.5)	0.909
Cesarean section	265 (60.6)	509 (58.2)	0.404
Other factors			
Apgar ≤7 at 1 min	140 (32.0)	335 (38.3)	0.025
Apgar ≤7 at 5 min	33 (7.6)	112 (12.8)	0.004
Required invasive ventilation	206 (47.1)	465 (53.2)	0.038
Duration of invasive ventilation, days	0 (0–3.0)	0.9 (0–4.0)	0.879
Required non-invasive ventilation	416 (95.2)	838 (95.9)	0.566
Duration of non-invasive ventilation, days	15.0 (6.0–29.0)	16.0 (6.0–29.0)	0.442
Total duration of ventilation, days	17.0 (7.0–33.0)	19.0 (7.0–33.0)	0.552
Length of hospitalization, days	49.24 ± 19.91	50.74 ± 19.83	0.198
Corrected age at discharge, weeks	36.77 ± 1.90	36.97 ± 1.87	0.075

HGP, hyperglycemia in pregnancy; HDP, hypertensive disorders of pregnancy. Values are mean ± SD, median (IQR) or frequency (%).

It was very interesting that fewer infants in the HGP group had low Apgar scores (≤7) both at 1 min and 5 min after matching (*P *< 0.05). Moreover, fewer infants in the HGP group required invasive ventilation after matching (47.1% *vs.* 53.2%, *P *= 0.038). No significant difference was found between the two groups in the median duration of invasive ventilation, the percentage of infants requiring noninvasive ventilation, the median duration of noninvasive ventilation, the median total duration of ventilation, or the mean corrected age at discharge after matching.

### 3.2 The impact of HGP on neonatal complications during hospitalization

To clarify the impact of HGP on the short-term outcomes of VPIs, we summarized the main complications of the hospitalized infants and made comparisons between the two groups. After matching, no difference was noted between the two groups in the total incidence of infants with RDS (total RDS and severe RDS), BPD (total BPD and moderate to severe BPD), NEC (NEC with Bell stage ≥Ⅱa and NEC surgery), ROP (total ROP and ROP treatment), PDA (total PDA diagnosed after 72 h and PDA treatment), IVH (total IVH and severe IVH), culture-positive sepsis (EOS and LOS), anemia (total anemia and anemia requiring red blood cell transfusion), PVL, FI, MBDP or PNAC. These are shown in Table 2.

**Table 2:** The neonatal complications during hospitalization between the HGP group and the non-HGP group.

Complications	HGP (No. = 437)	Non-HGP (No. = 874)	*P* value
RDS			
Total	309 (70.7)	597 (68.3)	0.375
Severe (grade 3–4)	57 (13.0)	150 (17.2)	0.054
BPD			
Total	214 (49.0)	434 (49.7)	0.815
Moderate to severe	74 (16.9)	161 (18.4)	0.508
NEC			
Bell stage ≥Ⅱa	43 (9.8)	69 (7.9)	0.235
Surgery NEC	6 (1.4)	13 (1.5)	0.870
ROP			
Total	146 (33.4)	265 (30.3)	0.256
Treated ROP	14 (3.2)	23 (2.6)	0.555
PDA			
Diagnosed after 72 h	237 (54.2)	466 (53.3)	0.754
Treated PDA	153 (35.0)	296 (33.9)	0.681
IVH			
Total	164 (37.5)	316 (36.2)	0.627
Severe (grade 3–4)	9 (2.1)	18 (2.1)	>0.999
Culture positive sepsis			
Early onset	15 (3.4)	16 (1.8)	0.072
Late onset	38 (8.7)	66 (7.6)	0.470
Anemia			
Total	365 (83.5)	745 (85.2)	0.416
RBC transfusion	254 (58.1)	545 (62.4)	0.139
PVL	29 (6.6)	47 (5.4)	0.358
Feeding intolerance	174 (39.8)	312 (35.7)	0.146
MBDP	13 (3.0)	24 (2.7)	0.814
PNAC	40 (9.2)	102 (11.7)	0.167

HGP, hyperglycemia in pregnancy; RDS, respiratory distress syndrome; BPD, bronchopulmonary dysplasia; NEC, necrotizing enterocolitis; ROP, retinopathy of prematurity; IVH, intraventricular hemorrhage; PDA, patent ductus arteriosus; RBC, red blood cells; PVL, periventricular leukomalacia; MBDP, metabolic bone disease of prematurity; PNAC, parenteral nutrition-associated cholestasis. Values are frequency (%).

### 3.3 The effect of HGP on fetal and neonatal growth

Birth weight, length, and head circumference can reflect fetal growth. After matching, the mean birth weight, length, and head circumference were similar between the two groups, as well as the incidence of IUGR respectively.

During hospitalization, there was no significant difference in the percentile of weight loss, age at birth weight recovery, weight gained per day after birth weight recovery, length or head circumference increased per week between the HGP group and the non-HGP group after matching.

At discharge or at 36 weeks of corrected age, a significant difference was noted only in EUGR for weight and head circumference (44.5% *vs.* 36.2%, *P *= 0.004; 35.5% *vs.* 28.6%, *P *= 0.013), not for length after matching.

In addition, we calculated the percentage of infants with increased growth retardation (IGR) from birth to discharge or at 36 weeks of corrected age. After matching, the percentages of IGR for weight and head circumference, but not for length, in the non-HGP group were higher than those in the HGP group (40.7% *vs.* 33.6%, *P *= 0.013; 26.4% *vs.* 20.4%, *P *= 0.016). All these are shown in Table 3.

**Table 3:** The fetal and neonatal growth between the HGP group and the non-HGP group.

Factors	HGP (No. = 437)	Non-HGP (No. = 874)	*P* value
At birth			
Birth weight, gram	1,360 ± 317	1,329 ± 293	0.081
Body length, cm	38.4 ± 3.2	38.3 ± 3.3	0.789
Head circumference, cm	27.3 ± 2.0	27.1 ± 2.1	0.283
IUGR for birth weight	11 (2.5)	33 (3.8)	0.233
IUGR for body length	40 (9.2)	96 (11.0)	0.305
IUGR for head circumference	36 (8.2)	79 (9.0)	0.629
Growth rate during hospitalization			
Percentile of weight loss, %	6.4 ± 4.3	6.6 ± 4.2	0.514
Age at birth weight recovery, days	9.4 ± 5.9	9.1 ± 4.3	0.387
Weight gained after recovering birth weight, grams/(kg.d)	16.1 ± 5.2	16.5 ± 6.2	0.227
Body length, cm/week	0.93 ± 0.38	0.90 ± 0.41	0.201
Head circumference, cm/week	0.62 ± 0.26	0.63 ± 0.27	0.935
At discharge or at 36 weeks of corrected age			
Body weight, gram	2,351 ± 350	2,338 ± 390	0.534
Body length, cm	45.0 ± 2.3	45.0 ± 2.6	0.958
Head circumference, cm	31.7 ± 1.4	31.7 ± 1.6	0.435
EUGR for body weight	158 (36.2)	389 (44.5)	0.004
EUGR for body length	162 (37.1)	370 (42.3)	0.067
EUGR for head circumference	125 (28.6)	310 (35.5)	0.013
IGR from birth to discharge or 36 weeks of corrected age			
IGR for body weight	147 (33.6)	356 (40.7)	0.013
IGR for body length	122 (27.9)	274 (31.4)	0.202
IGR for head circumference	89 (20.4)	231 (26.4)	0.016

HGP, hyperglycemia in pregnancy; IUGR, intrauterine growth retardation; EUGR, extrauterine growth retardation.

IGR, increased growth retardation. Values are mean ± SD or frequency (%).

### 3.4 Nutritional management during hospitalization

Nutrition intake is very important for infant growth. In this study, we also analyzed the nutritional management of the infants during hospitalization (Table 4). After matching, the initial time of enteral feeding in the non-HGP group was slightly later than that in the HGP group (24.0 (9.0–44.0) *vs.* 20.5 (5.0–30.0) h, *P *= 0.009). In addition, cumulative fasting time and age at reaching the oral calorie intake target were also longer in the non-HGP group than in the HGP group after matching (24.0 (17.0–34.0) *vs.* 23.0 (15.0–32.0) d, *P *< 0.05). After matching, age at achieving total enteral feeding, duration of parenteral nutrition, cumulative calories in the first week of hospitalization, age at reaching total calorie intake target, cumulative amino acid dose in the first week and during the whole hospitalization period, cumulative dose of fat emulsion in the first week and during the whole hospitalization period, were all similar between the two groups.

**Table 4:** Comparisons of nutritional intake during hospitalization between the HGP group and the non-HGP group.

Factors	HGP (No. = 437)	Non-HGP (No. = 874)	*P* value
Time to start enteral feeding, hours	20.5 (5.0–30.0)	24.0 (9.0–44.0)	0.009
Age at achieving total enteral feeding, days	25.0 (16.0–35.0)	26.0 (18.0–37.0)	0.068
Duration of parenteral nutrition, days	21.0 (12.0–32.0)	21.0 (14.0–31.5)	0.151
Cumulative fasting time, days	1.5 (0.7–5.0)	2.0 (1.0–5.0)	0.035
Cumulative calories in the 1st week of hospitalization, kcal/kg	505 (430–577)	496 (419–562)	0.074
Age at reaching total calorie intake target, days	9.0 (6.0–15.0)	9.0 (7.0–15.0)	0.123
Age at reaching oral calorie intake target, days	23.0 (15.0–32.0)	24.0 (17.0–34.0)	0.044
Cumulative amino acid dose in the 1st week of hospitalization, g/kg	16.0 (12.9–18.5)	16.1 (13.4–18.5)	0.320
Cumulative amino acid dose during hospitalization, g/kg	45.4 (24.0–75.5)	46.0 (27.0–74.3)	0.587
Cumulative dose of fat emulsion in the 1st week of hospitalization, g/kg	12.7 (9.7–15.0)	12.5 (10.0–15.0)	0.763
Cumulative dose of fat emulsion during hospitalization, g/kg	37.6 (19.4–63.2)	38.5 (21.3–62.6)	0.592

HGP, hyperglycemia in pregnancy; Values are median (IQR).

## 4 Discussion

In this study, we identified that HGP did not worsen the short-term outcomes of the surviving VPIs. Specifically, the VPIs born to mothers with HGP did not have a higher risk of main complications, including RDS, BPD, NEC, ROP, PDA, IVH, PVL, culture-positive sepsis, anemia, FI, MBDP or PNAC. In addition, these infants had a lower incidence of EUGR for weight and head circumference at discharge or at 36 weeks of corrected age, as well as less IGR for weight and head circumference during hospitalization.

To date, only a few studies have explored the association between HGP and short-term outcomes in VPIs or very low birth weight infants (8–13, 21). The study results were consistent regarding the impact of HGP on the increased risk of RDS, BPD, severe IVH, PDA requiring treatment, PVL and birth asphyxia but were conflicting regarding NEC (8, 9) and ROP (10). These inconsistent results may be due to the poor control of confounding factors and the difference in the inclusion criteria and subjects. To minimize the confounding effect, we conducted a 1:2 matched analysis by PSM with a nearest-neighbor matching algorithm to adjust for the six covariates, including gestational age, sex, antenatal steroid administration, delivery mode, twin or multiple births, and maternal gestational hypertension. After matching, we did not find a significant difference in the occurrence of RDS, BPD, PDA requiring treatment, EOS, IVH or PVL between the HGP group and the non-HGP group, which is consistent with the studies mentioned above (11–13).

RDS has been reported to increase in infants born to mothers with GDM (2). In recent years, there were three meta-analysis studies referring to mothers with GDM and neonatal complications. One study was published in 2019 and noted that the odds ratio of RDS was higher in women with GDM and insulin use (22). The other two studies were published in 2022 and pointed out that regardless of the GDM screening criteria, the risk of RDS significantly increased in women with GDM compared with the non-GDM group (23, 24). The populations involved in all the above meta-analysis studies included term and preterm infants. In the survival VPIs, we did not find that HGP increased the risk of RDS. Prematurity is the chief cause of RDS, and the risk for developing RDS is negatively correlated with gestational age (25). Surfactant appears at approximately 24 weeks of gestation in the cytosol of type II pneumocytes and increases with gestational age. It is not measurable in the amniotic fluid until approximately 32 weeks (26). Insulin can inhibit the secretion of glucocorticoids, which can accelerate fetal lung maturation. Women with HGP always have hyperinsulinemia, which delays fetal lung maturation and partly contributes to RDS. In our study, because the VPIs had a high incidence of RDS, the statistical effect of GDM on lung maturation might be weakened.

Consistent with the studies of Persson M et al. and Razak A et al. (11, 13), our study found that the risk of NEC in the surviving VPIs born to mothers with HGP did not increase when compared with the non-HGP group. However, two other studies reported that maternal diabetes was a risk factor for NEC in preterm infants. One was from Latin America (9), and they examined the in-hospital mortality and morbidity in very low birth weight infants born to mothers with and without diabetes mellitus. After logistic regression analysis, they found that NEC (grades 2–3) was the only condition independently associated with diabetes mellitus. Another study was from the Eunice Kennedy Shriver National Institute of Child Health and Human Development Neonatal Research Network (8). They examined morbidity in infants born at 22–28 weeks of gestation to mothers with insulin-dependent diabetes. The results showed that the infants born to mothers with insulin use before pregnancy had a higher risk of NEC; however, in this study, the incidence of maternal hypertension was also higher in these infants, which was found to be associated with NEC among very low birth weight infants (27).

ROP remains one of the predominant causes of blindness in preterm infants. The incidence varies with gestational age and birth weight (28). Several studies have assessed the other risk factors for severe ROP, including sepsis, RDS, BPD, PDA, neonatal hyperglycemia, blood transfusion, supplemental oxygen administration, mechanical ventilation, and preeclampsia (29–31). Opara CN et al. (10) found that maternal diabetes was associated with ROP and that the strength of the association increased with increasing ROP severity in very low birth weight infants, but the baseline characteristics, including gestational age, birth weight, neonatal steroid use and sepsis, were significantly different. In our study, after matching the confounding covariates in the baseline characteristics between the two groups, we did not find a significant association between ROP and HGP in VPIs. This is similar to other studies (8–13).

In mothers with HGP, intrauterine hyperglycemia and hyperinsulinemia can affect fetal growth, and the impact can last for some time after birth. In our study, the incidence of IUGR at birth was low in VPIs and were comparable between the HGP group and the non-HGP group after matching. At discharge or at 36 weeks of corrected age, the incidence of EUGR became high. These results were similar to those of previous studies (32, 33). In addition, we found that the VPIs in the HGP group had a lower rate of EUGR for weight and head circumference, as well as less IGR during hospitalization. Although the clear mechanisms are still unknown, there are some possible explanations in previous reports. Animal experiments found that rats exposed to maternal diabetes during pregnancy exhibited hyperinsulinemia, which could accelerate physical growth by increasing the storage of fat and protein. Other studies reported that exposure to human milk from mothers with HGP could change hypothalamic function, which might affect the satiety center and the regulation of body weight and metabolism (34, 35). Interestingly, the metabolic state of germ-free mice receiving meconium from VPIs changed significantly with a reduction in the plasma levels of insulin and leptin and resulted in obvious growth restriction. This suggested that the unique microbiota in VPIs may be prone to growth failure (36). However, Ting Chen (37) et al. found that the richness and diversity of the gut microbiota in mothers with HGP decreased, and the pathways related to carbohydrate and nucleotide metabolism were enriched, suggesting that maternal HGP may promote the growth of high-energy-supplying microbiota by promoting succession and thereby alter the metabolism of offspring. On the other hand, postnatal nutrition intake is very important to the growth of VPIs. Delayed enteral feeding and increased fasting time are associated with EUGR (38, 39). In our study, although there was no significant difference between the two groups in the duration of parenteral nutrition or age at reaching the total calorie intake target, the initial time of enteral feeding in the non-HGP group was later than that in the HGP group. Moreover, the cumulative fasting time and age at reaching the oral calorie intake target were longer in the non-HGP group than in the HGP group.

## 5 Conclusions

In conclusion, our study found that HGP did not worsen the short-term outcomes of surviving VPIs, as it did not lead to a higher risk of the main neonatal complications and even led to improved growth during hospitalization. However, there are still some limitations in our study. First, the VPIs eligible for analyses were only survivors and were not based on the whole population. Second, more details, including the pattern of HGP (GDM or PGDM) and the treatment of HGP before delivery, could not be analyzed. Further studies in the future are needed.

## Data Availability

The raw data supporting the conclusions of this article will be made available by the authors, without undue reservation.
